# Activation of SGK1/ENaC Signaling Pathway Improves the Level of Decidualization in Unexplained Recurrent Spontaneous Abortion

**DOI:** 10.1007/s43032-023-01273-1

**Published:** 2023-06-06

**Authors:** Xiaoqian Di, Yanzhi Hao, Zibo Duan, Yucong Ma, Ying Cao, Zhanwang Tan, Cuimiao Song, Xiaohua Lin

**Affiliations:** 1 Hebei University of Chinese Medicine, Shijiazhuang, 050091 Hebei China; 2 Collaborative Innovation Center of Integrated Chinese and Western Medicine On Reproductive Disease, Hebei University of Chinese Medicine, Shijiazhuang, 050091 Hebei China; 3 Hebei Key Laboratory of Integrative Medicine On Liver-Kidney Patterns, Hebei University of Chinese Medicine, Shijiazhuang, 050091 Hebei China; 4 North China University of Science and Technology, Shijiazhuang, 050011 Hebei China; 5 Hebei Medical University, Shijiazhuang, 050011 Hebei China; 6 Hebei Hospital of Traditional Chinese Medicine: Affiliated Hospital of Hebei University of Chinese Medicine, Shijiazhuang, 050011 Hebei China

**Keywords:** URSA, Estrogen and progesterone, SGK1/ENaC, Decidualization

## Abstract

Recurrent spontaneous abortion (RSA) is one of the most common complications during pregnancy and seriously affects women's physical and mental health. About 50% of RSA cases are of unknown etiology. Our previous study found that the decidual tissue of patients with unexplained recurrent spontaneous abortion (URSA) had low expression levels of serum and glucocorticoid-induced protein kinase (SGK) 1. Endometrial decidualization is a key link in the early stage of pregnancy and is crucial to the development and maintenance of pregnancy. Decidualization is the proliferation and differentiation of endometrial stromal cells into deciduals, which involves a complex physiological process such as ovarian steroid hormones (estrogen, progesterone, prolactin, etc.), growth factors, and intercellular signaling. The binding of estrogen and its receptor stimulates the synthesis of endometrial deciduating markers prolactin (PRL) and insulin-like growth factor binding protein 1 (IGFBP-1), which mediates the occurrence of decidualization. Among them, SGK1/ENaC is a signaling pathway closely related to decidualization. The purpose of this study was to further investigate the expression of SGK1 and decidualization-related molecules in the decidual tissue of URSA patients and to explore the potential mechanism of SGK1’s protective effect in URSA patients and in mouse models. Decidual tissue samples from 30 URSA patients and 30 women who actively terminated pregnancy were collected, and a URSA mouse model was established and treated with dydrogesterone. Expression levels of SGK1 and its signaling pathway-related proteins (p-Nedd4-2, 14–3-3 protein and ENaC-a), estrogen and progesterone receptors (ERβ, PR), and decidualization markers (PRLR, IGFBP-1) were assessed. Our study found that SGK1, p-Nedd4-2, 14–3-3 proteins, and ENaC-a expression levels were reduced in the decidual tissue, the SGK1/ENaC signaling pathway was inhibited, and the expression levels of the decidualization markers PRLR and IGFBP-1 were downregulated in the URSA group compared with the controls. Additionally, the concentrations of E_2_, P, and PRL in the serum of mice were decreased in the URSA group compared with the controls. However, SGK1/ENaC pathway-related proteins, estrogen and progesterone and their receptors, and decidualization-related molecules were upregulated by dydrogesterone. These data suggest that estrogen and progesterone can induce decidualization by activating the SGK1/ENaC signaling pathway; disruption of this pathway can lead to the development of URSA. Dydrogesterone can increase the expression level of SGK1 protein in decidual tissue.

## Introduction

The loss of the product of two consecutive or discontinuous pregnancies with a same-sex partner before 24 weeks is called recurrent spontaneous abortion (RSA) or recurrent pregnancy loss (RPL) [1, 2]. The etiology of the disease is very complex; while chromosomal factors, anatomical factors, hormonal problems, infections, endocrine anomalies, antiphospholipid syndrome, and vascular thromboembolic diseases are accepted etiologic causes, the etiology of the disease is still unknown in about 50% of RSA patients [3]. The clinical diagnosis in these patients is unexplained recurrent spontaneous abortion (URSA) [4]. In recent years, the incidence of recurrent miscarriage has risen markedly to about 10% of married women of reproductive age [5]. At present, the occurrence of 2 consecutive miscarriages should be taken seriously and evaluated because the risk of two miscarriages is similar to that of 3 miscarriages, about 1 to 3% [6]. The prognosis worsens with increased age [7]. RSA, which has become a hot research topic, causes patients and their families to suffer huge physical and mental harm and economic losses. Impaired endometrial decidualization may play an important role in the pathogenesis of URSA [8].

Serum and glucocorticoid-induced protein kinase (SGK) 1, a member of the serine/threonine protein kinase family, was discovered in rat mammary tumor cells by Webster et al. [9]. Recent studies have shown that SGK1 plays important roles in promoting cell survival and regulating ion channels. Estrogen receptor beta (ERβ) is enriched at the SGK1 promoter, and estrogen directly increases SGK1 transcription by upregulating ERβ [10]. When progesterone levels rise, SGK1 responds quickly. Under physiological conditions, the expression of SGK1 on the decidua precedes pregnancy, and it increases sharply in the mid-secretory stage and is maintained until the late-secretory stage [11], showing a periodic dynamic expression pattern. As pregnancy progresses, SGK1 expression further increases in the decidua and villous tissue at the maternal–fetal interface [12]. Low levels of SGK1 in the endometrium are associated with pregnancy failure [10]. A recent study found that the expression of SGK1 in the decidua and villi at the maternal–fetal interface in RSA patients was lower than that in patients with normal pregnancies [13]. In addition, animal experiments have also shown that blocking the gene expression of SGK1 in mice to reduce SGK1 levels can lead to miscarriage [14]. Importantly, these studies showed that during the implantation window there must be a decline in SGK1 expression levels in luminal epithelium to infer endometrial receptivity. Further, to investigate if sustained activity of SGK1 in the endometrial epithelium interferes with embryo implantation, an expression vector encoding constitutively active SGK1 (SGK-S422D) was injected into the uterine lumen of wild-type female mice (using a natural mating model). The results showed a complete abolishment of implantation sites in animals expressing constitutively active SGK1 [15]. Furthermore, aberrant expression of SGK1 in the mouse luminal epithelium either suppressed or completely abolished the induction of key endometrial receptivity markers, such as leukemia inhibitory factor (Lif), heparin-binding EGF-like growth factor (Hbef) and homeobox protein Hox-A10 (Hoxa10) leading to conception delay and thus implantation failure [16]. Fisher and Giudice revealed that in pregnant Sgk1 knockout (sgk1^–^/^–^) mice, the uteri/implantation sites, showed evidence of uterine bleeding, deficiency in fetal growth and spontaneous (30%) fetal loss akin to human miscarriage [17].

Epithelial sodium channels (ENaCs) are amiloride-sensitive sodium channels located in the apical membrane of various epithelia, expressed in both human and mouse endometrial epithelium, and represent one of the important downstream targets of SGK1 [18]. Stimulation of SGK1 is a common pathway for regulating ENaC activity. SGK1 can promote the binding of phospho-Nedd4-2 to 14–3-3, resulting in a change in the conformation of Nedd4-2, thereby preventing the ubiquitination of ENaC by Nedd4-2 and increasing ENaC on the cell membrane [19]. ENaC-a plays a key role in translating signals from the implanted embryo to downstream cellular responses, leading to decidualization of the matrix [20]. Previous studies have shown that the expression of ENaC-a in the decidua decreased in both mouse abortion models and clinical abortion cases, suggesting that ENaC-a may play an important pathophysiological role in the post-implantation decidua [21].

Decidualized stromal cells transform from fibroblasts to epithelioid cells and secrete the decidualization hallmark hormones prolactin (PRL) and insulin-like growth factor binding protein-1 (IGFBP-1) [22]. The process of decidualization is regulated by multiple endocrine, paracrine and autocrine factors, among which 17β-estradiol and progesterone play important roles [23]. However, the mechanism of action of the estrogen- and progesterone-mediated SGK1/ENaC signaling pathway in URSA patients has received less attention. In addition, given that low progesterone-related luteal insufficiency is the main cause of embryo implantation failure and early embryo loss, routine clinical supplementation of progesterone to URSA patients can prevent miscarriage and prolong gestational age [24]. However, the exact mechanism is still unclear. Therefore, this study aimed to explore the mechanism of SGK1 signaling in URSA and the effect of the activation of SGK1 on URSA in order to provide a theoretical basis for the clinical treatment of URSA patients.

## Materials and Methods

### Study Participants

From January 2022 to September 2022, this study recruited 60 pregnant women under the age of 40 with a history of two or more unexplained miscarriages from the Obstetrics and Gynecology Department of Hebei Provincial Hospital of Traditional Chinese Medicine. The exclusion criteria included abnormal menstrual cycle, genital infection, abnormal uterine cavity, antiphospholipid syndrome, thrombotic disease, chronic hypertension, diabetes, kidney disease, thyroid disease, autoimmune disease, and cardiovascular disease as well as chromosomal abnormalities in either spouse. The pregnant women underwent transvaginal ultrasonography every 2 weeks. When the fetal heart rate was found to be absent, it was defined as pregnancy termination. Chorionic villus sampling was also performed for chromosome testing. Decidual tissue during induced abortion was collected, and after excluding patients with chromosomal abnormalities in the decidual tissue, only 30 patients were recruited for follow-up study. The control group included 30 pregnant women from the same hospital who had elected to terminate their pregnancy through abortion, and decidua tissue was collected from them. The biological sample study was approved by the Medical Ethics Committee of the First Affiliated Hospital of Hebei University of Traditional Chinese Medicine (ethics approval number: HBZY2021-KY-133–01, China).

### Experimental Animal

Female CBA/J mice, male DBA/2 mice, and male BALB/c mice (8–10 weeks old) were purchased from Beijing Huafukang Biotechnology Co., Ltd. The mice were adaptively fed autoclaved sterile water and chow for one week in a standard environment with a constant temperature of 21–23 °C and a humidity of 50–60%. The animal experiments were performed under specific sterile pathogen conditions in the Experimental Animal Center of Hebei University of Traditional Chinese Medicine. All experimental protocols were approved by the Experimental Animal Ethics Committee of Hebei University of Traditional Chinese Medicine (ethics number: DWLL202208003, China) and are in accordance with the Guide for the Care and Use of Laboratory Animals (National Academy of Sciences, Copyright 2010).

### Establishment and Intervention of URSA Mouse Model

The female mice were randomly divided into three groups. Female CBA/J mice were mated with male BALB/c mice as the control group, and female CBA/J mice were mated with male DBA/2 mice as the URSA group. Dydrogesterone (Abbott Trading Co., Ltd., China)-treated female CBA/J mice were mated with male DBA/2 mice for the dydrogesterone-treated group (URSA + DQYT). The female mice in the dydrogesterone-treated group were given dydrogesterone (0.5 mg/kg) by gavage every day 14 days before mating and after seeing vaginal suppositories, while the control and URSA groups were given the same content of normal saline by gavage. For successful mating, two females and one male were co-housed overnight, and females with vaginal plugs detected the next morning were recorded as gestational day (GD) 1. The food intake, activity level, vaginal bleeding and body weight of the mice in each group were observed daily. Female mice were euthanized on day 8 GD. The uterus and decidua were separated, and all tissues were fixed with 4% paraformaldehyde and stored at -80 °C. At the same time, the total number of embryos and the number of viable embryos were calculated. The embryos of the mice were small in size and black in color, and part of the mouse uterus showed bamboo-like changes with necrosis and hemorrhage and lost embryos. The embryo loss rate is calculated as the number of embryos lost/total number of embryos X100%.

### ELISA

Bleeding was performed by enucleating mouse eyeballs. The blood samples were allowed to stand at room temperature for 2 h and then centrifuged at 3000 rpm for 15 min, and then the supernatant was removed and stored at − 80 °C. ELISA kits (Tianjin Xiehe Pharmaceutical Technology Group Co., Ltd.) were used to detect the concentrations of E_2_, P, and PRL in the serum. The experimental steps were carried out in strict accordance with the instructions.

### HE Staining

The mouse decidual tissue was fixed with 4% paraformaldehyde for 24–48 h, and then the samples were dehydrated with graded ethanol, paraffin embedded, and histologically sectioned. The paraffin sections were dewaxed with xylene and ethanol, stained with hematoxylin for 3 min and eosin for 2 min, and then mounted with neutral resin. The morphological changes of decidua were observed under an inverted microscope, and pathological pictures were taken.

### Immunohistochemistry

The paraffin sections of decidual tissue were obtained on day GD8. The tissue sections were deparaffinized with xylene and graded ethanol after baking in a 60 °C oven for 2 h. The sections were then penetrated with 2% Triton solution for 20 min and repaired with sodium citrate antigen retrieval solution. After blocking with ordinary goat serum for 30 min, the sections were incubated with SGK1 (diluted, 1:1600), ENaC-a (diluted, 1:800), ERβ (diluted, 1:800), or PR (diluted, 1:1600) primary antibodies at 4 °C overnight and then incubated with biotin-labeled anti-rabbit IgG (1:1 000) for 2 h at room temperature. After washing, the samples were incubated with horseradish peroxidase-labeled streptavidin working solution for 30 min, developed with DAB, and counterstained with hematoxylin. After mounting with neutral resin, the positive cells were observed under a Zeiss microscope (Carl Zeiss AG, Oberkochen, Germany).

### Western Blot Assay

The mouse decidua tissue was extracted with lysis buffer, and the protein concentration was detected with a BCA protein quantification kit (Beyotime, Shanghai, China). The total lysates were separated by 10–12% sodium dodecyl sulfate–polyacrylamide gel electrophoresis (SDS-PAGE) and transferred to polyvinylidene fluoride membranes (PVDF). After blocking with 5% skim milk for 2 h, the membranes were incubated with SGK1 primary antibody (diluted, 1:1000), phospho-Nedd4-2 (phospho S448, diluted, 1:1000), washed overnight at 4 °C, and then incubated with secondary antibody for 2 h at room temperature. An ECL detection kit (New Saimei Biotechnology Co., Ltd.) was then used for signal detection. GAPDH (dilution, 1:2000) was used as an internal reference control.

### Real-time Fluorescence Quantitative PCR Detection

Total RNA from human and mouse decidual tissues was extracted using Trizol reagent (Promega, USA) according to the manufacturer’s instructions. Reverse transcription was performed using a GoScript Reverse Transcription System kit (Promega, USA). RT-PCR was performed using a 7500 detection system (model: 7500Fast, Thermo Scientific, USA) and a GoTaq qPCR Master Mix kit (Promega, USA). The relative quantification of target gene expression, that is, the RQ value, was calculated using the formula RQ = 2^−ΔΔct^. The primers were designed and synthesized by Beijing Servicebio Company, and the primer sequences are listed in Table 1.

**Table 1:** The primers used for quantitative real-time PCR

Gene	Amplified fragment length(bp)	Primer sequences(5′-3′)
H-GAPDH-S	121	GTCATTCCAAATATGAGATGCGT
H-GAPDH-A		GCTATCACCTCCCCTGTGTG
H-SGK1-S	115	GAAGGTGGATCAAGAGCCCAA
H-SGK1-A		GAAGACTCGAAGTCTGGCTCC
H-PRLR-S	105	TGGAGCTTCATGTCCTCGTG
H-PRLR-A		CTCAGTGTTCGCCTCCATGA
H-IGFBP-1-S	91	CCTTTGGGACGCCATCAGTA
H-IGFBP-1-A		GAGTTCTATTCGGCAGGGCT
M-SGK1-S	137	CATCACCTGGAGCAGAGCGA
M-SGK1-A		TGGCGGGATACAGCAAATCTA
M-PRLR-S	82	CAAGGATGCCTGCCTGGTTA
M-PRLR-A		TGAGCTGCTCCAAAACCACA
M-IGFBP-1-S	118	CTGTCCTTCCAGATTGGCGT
M-IGFBP-1-A		GAGAAATCTCGGGGCACGAA
M-14–3-3-S	92	GCCGAGCACTAAGACGACAT
M-14–3-3-A		ACCGGAAGCAGGTTATGCAA
M-ENaC-a-S	134	AGGACCCAGGAGGAGATAGGG
M-ENaC-a-A		CTGTTTGACTCTCCGGCTTTC
M-GAPDH-S	111	TTCACCACCATGGAGAAGGC
M-GAPDH-A		CTCGTGGTTCACACCCATCA

### Statistical Analysis

SPSS 26.0 ImageJ, and Image Pro Plus 6.0 software programs were used for the statistical analysis, the measurement data were $\overline{x }$±s, and the count data were analyzed by RXC cross-tabulation. The original data were first tested for normal distribution and homogeneity of variance. If they conformed to a normal distribution and homogeneity of variance, the comparison between the two groups of quantitative data was performed using the t test of independent sample means comparison. If the data confirmed to a normal distribution, a one-way ANOVA test was used to compare the means of multiple groups; an LSD test was used when the variance was homogeneous; Dunnett’s T3 test was used when the variance was unequal; and the disordered count data were compared using a χ^2^ test. *P* < 0.05 was considered to be statistically significant.

## Results

### Downregulation of SGK1 and Decidualization-Related Molecules in Decidual Tissue of URSA Patients

**Fig. 1:**
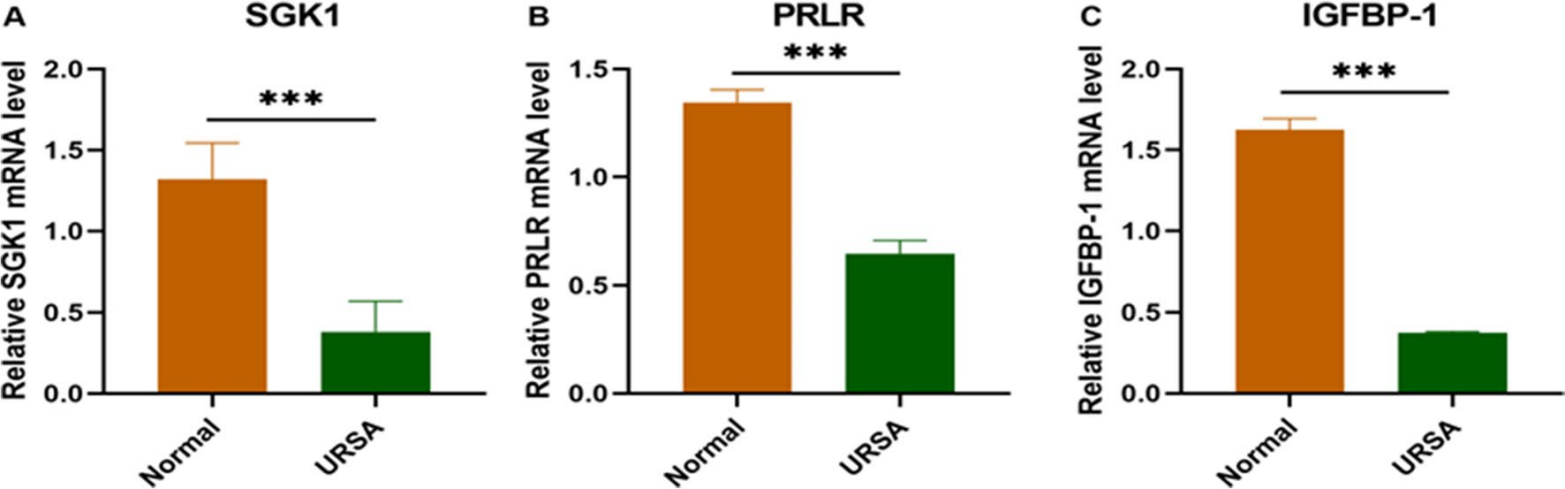
The expression of SGK1 and methylation-related molecule PRLR and IGFBP-1 in decidual tissue of URSA patients. **A** Expression of SGK1 mRNA in the URSA group and normal group by RT-PCR. **B** Expression of PRLR mRNA in the URSA group and normal group by RT-PCR. **C** Expression of IGFBP-1 mRNA in the URSA group and normal group by RT-PCR. All the results were representatives of three independent experiments and data were expressed as mean ± SD (*n* = 16/group). ***P < 0.001 compared to the control

Decidual tissue was obtained from patients to study the expression of SGK1 and decidualization-related molecules. The mRNA expression of SGK1 in the decidual tissue of URSA patients (URSA group) was lower than that of controls (normal group) (Fig. 1A), and the mRNA expression levels of PRLR and IGFBP-1 were also downregulated in the URSA group (Fig. 1B, C) (*P* < 0.001). The above results suggest that SGK1 and decidualization may be involved in URSA.

### Activation of SGK1 Reduces Embryo Loss in URSA Mice

**Fig. 2:**
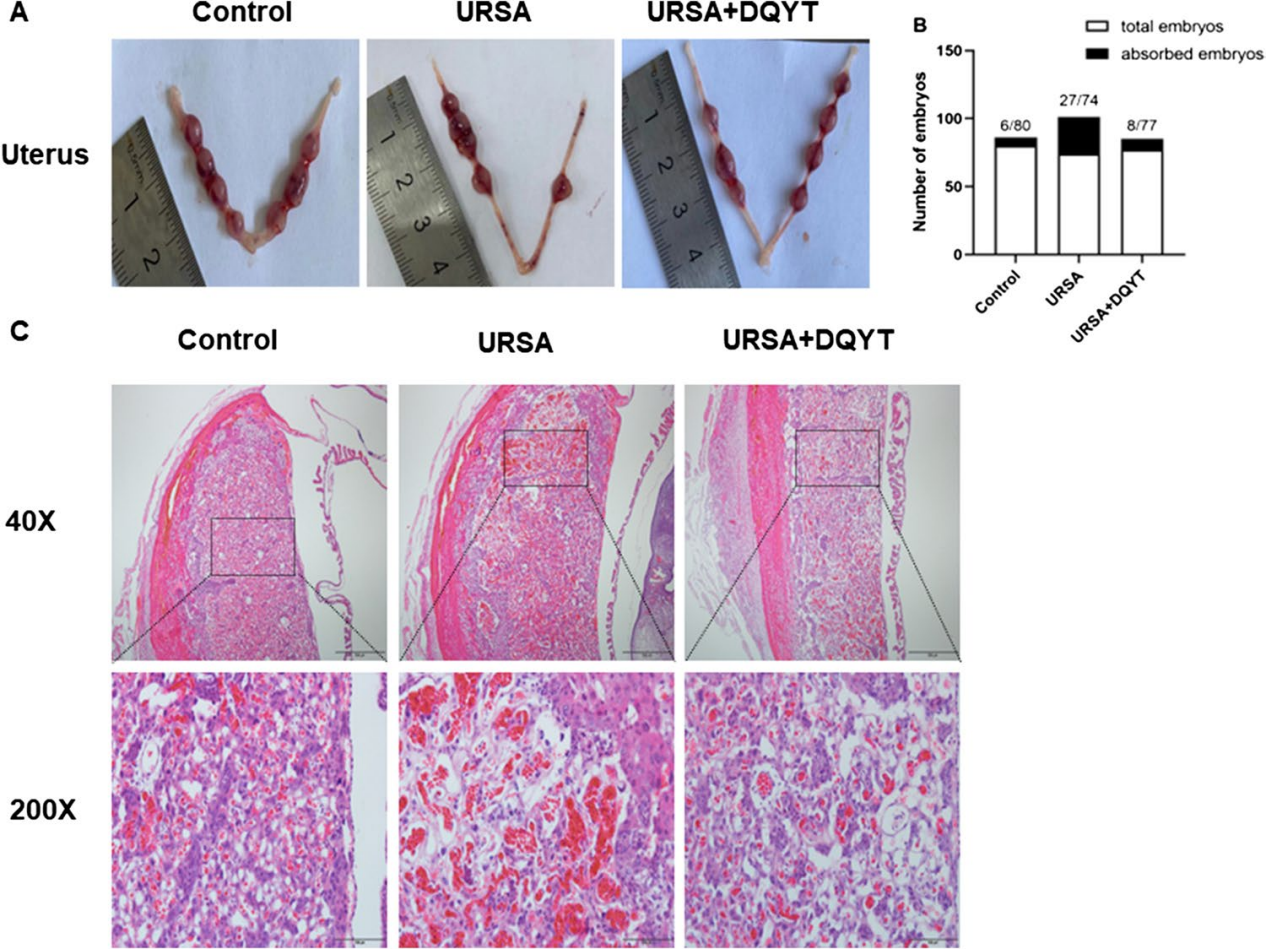
Uterus anatomy and pathological changes of decidua in mice. **A** Left and right uterine horns from normal pregnant mice, aborted mice, and drug-treated mice. **B** The number of fetuses. **C** Decidua of the control group, URSA group, and URSA + DQYT group were stained by H&E staining. Scale bar represents 500 µm and 100 µm (*n* = 4/group)

To study the pathogenesis of URSA, we established a URSA mouse model. The uteruses and embryos of the three groups of mice are shown in Fig. 2 A. Compared with the control group, some placentas and fetuses in the URSA group were smaller and darker in color. There were fewer miscarriages in the dydrogesterone-treated group than in the URSA group. The fetal numbers and embryo loss rates are shown in Fig. 2 B and Table 2. The embryo loss rate was 7.50% in the control group, 36.49% in the URSA group, and 10.39% in the dydrogesterone group. The differences in the embryo loss rates between the URSA and control groups (*P* < 0.001) and the URSA and dydrogesterone groups (*P* < 0.05) were statistically significant, indicating that the URSA mouse model was successfully established and dydrogesterone could reduce the embryo loss rate in the URSA group. Furthermore, the results of the HE staining showed that the uterine interstitium in the control group was loose and edema, showing decidualization and abundant blood vessels. In the URSA group, the thickness of the endometrium became thinner, the stroma was dense, and the number of blood vessels was reduced, accompanied by cell disorder and nuclear fragmentation. In the dydrogesterone group, the endometrium was thickened, and the expression of blood vessels in the interstitium was abundant (Fig. 2C).

**Table 2:** The embryo loss rate among the three groups (*n* = 10)

Group	Total embryos	Viable embryos	Lost embryos	Embryo loss rate (%)
Control	80	74	6	7.50% (6/80)
Model	74	47	27	36.49% (27/74)^a^
DQYT	77	69	8	10.39% (8/77)^b^

vs control group, ^a^*P* < 0.001; vs model group, ^b^*P* < 0.05

### Dydrogesterone Treatment Upregulates SGK1 Levels

**Fig. 3:**
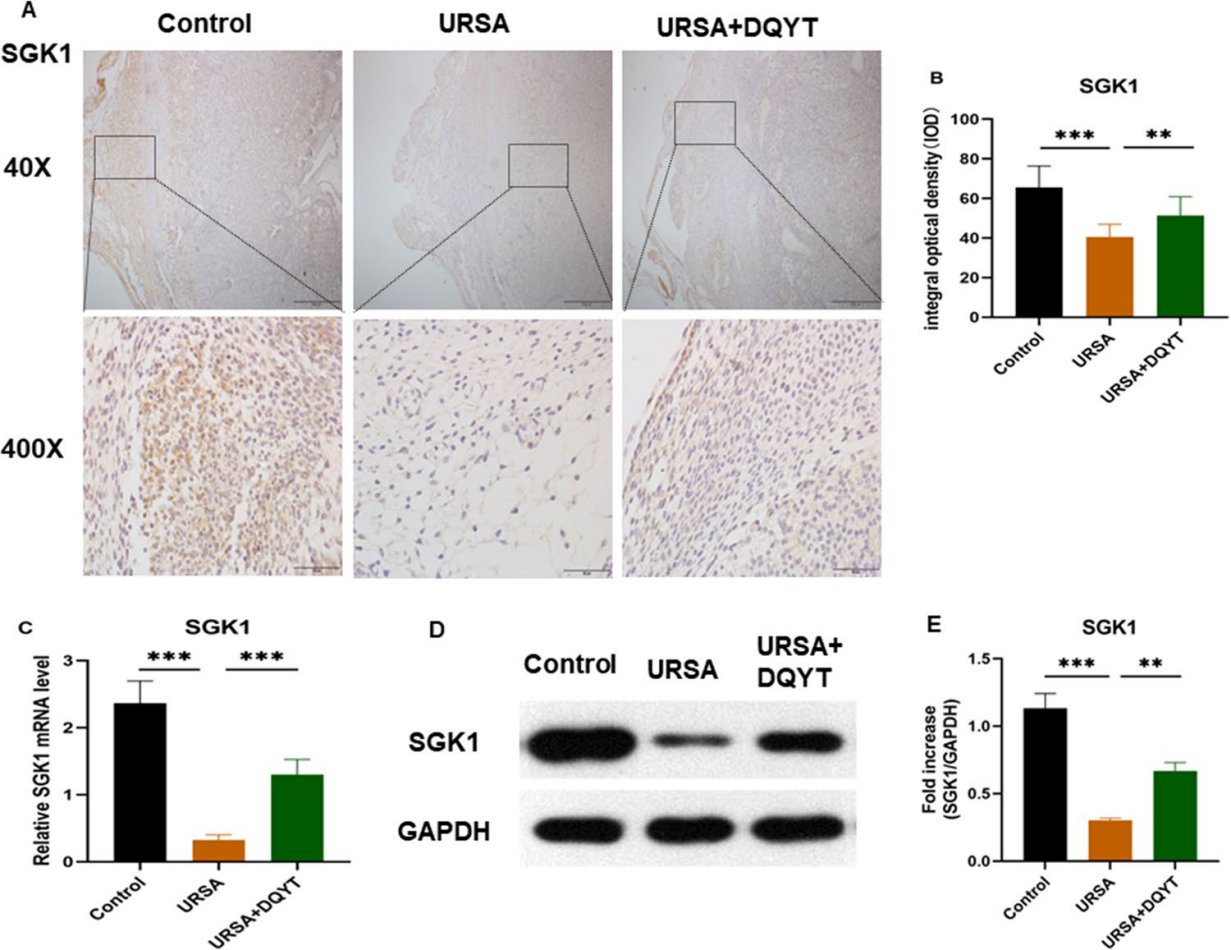
The expression of SGK1 in mouse decidual tissue. **A** The expression of SGK1 in mouse decidual tissue was investigated by immunohistochemistry staining. Scale bar represents 500 µm and 50 µm (*n* = 4/group). **B** Quantitative assessment of the SGK1 positive staining area in decidua using Image-Pro Plus software. **C** Expression of SGK1 mRNA in the control group, URSA group, and URSA + DQYT group by RT-PCR. (*n* = 8/group). **D** The protein levels of SGK1 were identified by Western blot analysis. **E** The quantification data analysis of SGK1 in the mouse decidual tissues (*n* = 3/group). All the results were representatives of three independent experiments and data were expressed as mean ± SD. **P < 0.01, ***P < 0.001

To verify the role of SGK1 in the URSA mouse model, we examined the expression of SGK1 in the decidual tissue of URSA mice by immunohistochemical staining. As shown in Fig. 3 A and B, the positive staining of SGK1 in the cytoplasm was significantly decreased in the URSA group compared with the control group (*P* < 0.01). In the dydrogesterone-treated group, the expression of SGK1 was increased compared with the URSA group. Real-time fluorescence quantitative PCR detection showed that the mRNA expression level of SGK1 in the URSA group was lower than that in the control group, but the level was increased in the dydrogesterone-treated group compared with the URSA group (Fig. 3C) (*P* < 0.001). Western blotting showed similar results: the expression level of SGK1 protein in the URSA group was lower than that in the control group, and the expression level was increased in the dydrogesterone-treated group (Fig. 3D, E) (*P* < 0.001). These results suggest that dydrogesterone treatment activates the expression of SGK1 in URSA mice.

### Alteration of SGK1 Signaling Pathway in Aborted Tissues of URSA Mice

**Fig. 4:**
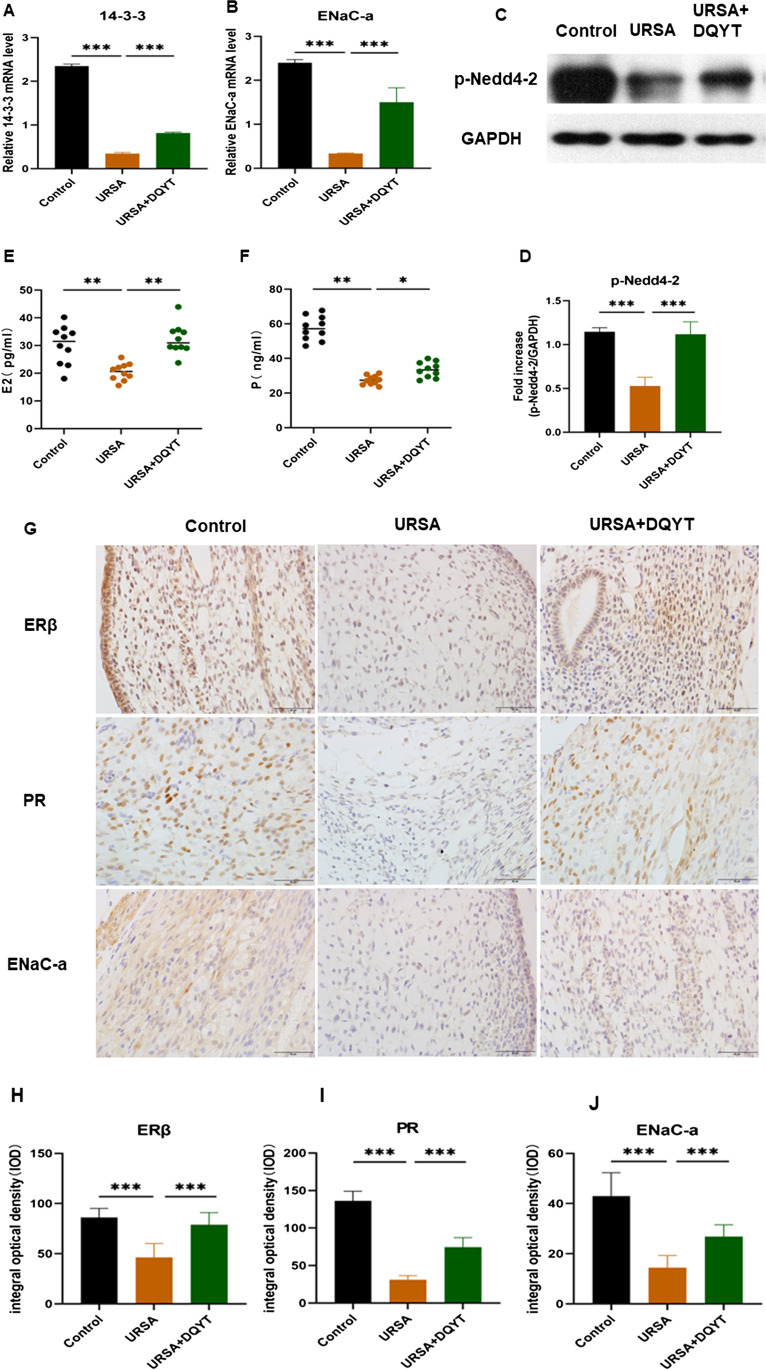
The analysis of SGK1 signaling pathway in mouse decidua and serum. **A** Expression of protein 14–3-3 mRNA in the control group, URSA group, and URSA + DQYT group by RT-PCR (*n* = 8/group). **B** Expression of ENaC-a mRNA in the control group, URSA group, and URSA + DQYT group by RT-PCR. (*n* = 8/group). **C** The protein levels of p-Nedd4-2 were identified by Western blot analysis. **D** The quantification data analysis of p-Nedd4-2 in the mouse decidual tissues. (*n* = 3/group). **E** The serum E_2_ levels of mice among control group, URSA group, and URSA + DQYT group were analyzed by ELISA (*n* = 10/group). **F** The serum P levels of mice among control group, URSA group, and URSA + DQYT group were analyzed by ELISA (*n* = 10/group). **G** The expression of ERβ, PR, AND ENaC-a was detected by immunohistochemistry in decidua among control group, URSA group, and URSA + DQYT group. Scale bar represents 50 µm (*n* = 4/group). **H** Quantitative data analysis for ERβ. **I** Quantitative data analysis for PR. **J** Quantitative data analysis for ENaC-a. All the results were representatives of three independent experiments and data were expressed as mean ± SD. *P < 0.05, **P < 0.01, ***P < 0.001

We examined the 14–3-3 and ENaC-a mRNA levels in the three groups of mice using real-time PCR. Compared with the control group, the 14–3-3 and ENaC-a mRNA expression levels were significantly downregulated in the URSA group, while the expression levels were significantly upregulated in the dydrogesterone group compared with the URSA group (Fig. 4A, B) (*P* < 0.01). Western blotting showed results: the expression level of p-Nedd4-2 protein in the URSA group was lower than that in the control group, and the expression level was increased in the dydrogesterone-treated group (Fig. 4C, D) (*P* < 0.001). At the same time, the serum E_2_ and P content of mice in the three groups was detected by ELISA (Fig. 4E, F). The results showed that the expression levels of serum E_2_ and P were lower in the URSA group than those observed in the control group but were significantly increased after dydrogesterone treatment (*P* < 0.05). Finally, the expression levels of ERβ, PR and ENaC-a in the three groups were detected by immunohistochemical staining. Low levels of ERβ and PR were mainly expressed in the nucleus of the decidual tissue of the URSA group and to a lesser extent in the cytoplasm, whereas ENaC-a was mainly expressed in the cytoplasm. The staining patterns in the dydrogesterone and control groups were similar to each other (Fig. 4G–J). Taken together, these results demonstrated that the SGK1 signaling pathway-related molecules (p-Nedd4-2/14–3-3/ENaC) were downregulated in the decidual tissue of the URSA group, suggesting that the SGK1/ENaC signaling pathway was inhibited, and its expression might be upregulated after dydrogesterone intervention. Therefore, we believe that the SGK1/ENaC pathway may play an important role in the decidual tissue of URSA, and estrogen and progesterone may activate this pathway.

### Activation of SGK1 Upregulates Decidualization-Related Molecules in Abortive Tissues of URSA

**Fig. 5:**
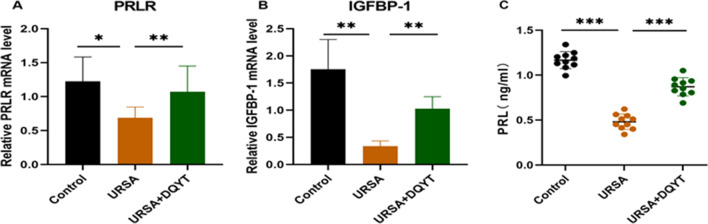
The analysis of PRLR, IGFBP-1, and PRL in mouse decidua and serum. **A** Expression of PRLR mRNA in the control group, URSA group, and URSA + DQYT group by RT-PCR (*n* = 8/group). **B** Expression of protein IGFBP-1 mRNA in the control group, URSA group, and URSA + DQYT group by RT-PCR (*n* = 8/group).** C** The serum PRL levels of mice among control group, URSA group, and URSA + DQYT group were analyzed by ELISA (*n* = 10/group). All the results were representatives of three independent experiments and data were expressed as mean ± SD. *P < 0.05, **P < 0.01, ***P < 0.001

To determine the expression of decidualization-related molecules in mouse tissues, we examined the mRNA expression of PRLR and IGFBP-1 in the three groups by real-time quantitative PCR (Fig. 5A, B). The results showed that the expression levels of PRLR and IGFBP-1 were consistent with those in human decidual tissue, and the expression levels in the URSA group were lower than those observed in the control group (*P* < 0.05). The expression levels were upregulated in the dydrogesterone-treated group compared with the URSA group (*P* < 0.05). The results of the mouse serum ELISA also showed that the level of PRL was decreased in the URSA group compared with the control group, but the level was then increased in the dydrogesterone group (*P* < 0.001) (Fig. 5C). These findings indicate that dydrogesterone could interfere with decidualization in URSA mice.

## Discussion

Endometrial decidualization is a key link in the establishment and maintenance of normal pregnancy. It is the terminal differentiation of endometrial stromal cells. This process involves massive cell proliferation, differentiation and apoptosis and is regulated by a complex molecular signaling network, essential for implantation of the embryo and establishment of late pregnancy [25]. The degree and quality of decidua have a significant impact on subsequent trophoblast invasion, placenta formation, and intrauterine growth. Abnormal decidualization can lead to repeated embryo loss [8], which is not necessarily related to whether the embryo is normal [26]. Studies have shown that approximately 30% of miscarriages occur in the first few months after implantation, and this early pregnancy failure is closely related to whether decidualization of stromal cells occurs normally at the time of embryo implantation [27]. It has been found that RSA patients have reduced transcript levels and activity of SGK1 in the secretory endometrium as well as p-SGK1 expression in stromal cells compared with healthy subjects [28]. Studies have also shown that SGK1 expression is reduced during the formation of the maternal–fetal interface in RSA patients [29]. Our experiments demonstrated that the mRNA expression level of SGK1 was downregulated in the decidual tissue of URSA patients compared with controls. In addition, we also found that the mRNAs of PRLR and IGFBP-1 were downregulated in the decidual tissues of URSA patients, suggesting that SGK1 and decidualizing factors play crucial roles in maintaining pregnancy, and their downregulation contributes to the development of URSA.

SGK1 is highly conserved and ubiquitously expressed in multiple cell types, including decidual stromal cells and trophoblast cells, and is considered an important node for signaling at the maternal–fetal interface in early pregnancy [16]. SGK1 is activated in response to various extracellular stimuli, such as insulin, growth factors, estrogen, and progesterone, suggesting a potential correlation between E_2_, P, and SGK1 at the maternal–fetal interface during the first trimester [30]. Chromatin immunoprecipitation analysis revealed that ERβ is enriched at the SGK1 promoter site 2- and 2.5-kb upstream of the SGK transcription start site [31], and it subsequently exerts biological functions such as promoting endometrial transformation and decidualization through gene transcription [32]. The expression of SGK1 is increased during the decidualization and secretion phases under the influence of progesterone [33]. Our study found that the expression of SGK1, ERβ and PR was low in the decidual tissue of mice in the URSA group. At the same time, the serum E_2_ and P levels of the mice in the URSA group were significantly decreased. However, after the intervention with dydrogesterone, the expression levels of SGK1, estrogen, and progesterone and their receptors were upregulated. In addition, the endometrial thickness of the mice in the URSA group was thinner, the interstitium was denser, and the number of blood vessels was reduced compared with the controls. These findings were accompanied by cell disorder and insufficient decidualization reaction. These data demonstrate that low levels of SGK1 are associated with impaired decidualization and that dydrogesterone can upregulate SGK1 expression levels.

Several studies have reported that SGK1 is involved in multiple processes in pregnancy, including maintaining endometrial receptivity, promoting blastocyst implantation and trophoblast invasion, and regulating decidual cell differentiation and proliferation and survival [34]. The ENaC protein is an ion channel, and previous studies have shown that the expression of ENaC-a correlates with placental function [35]. Activation of ENaC-a in the mouse endometrium is highest at implantation, and defective or low expression of ENaC-a in the endometrium may lead to lower success rates in in vitro fertilization (IVF) patients [36]. Many studies in the literature suggest that ENaC is regulated by a variety of factors, including hormones; proteases; other signaling molecules [37], such as SGK1; neural precursor cell-expressed developmentally downregulated 4–2 (Nedd4-2) [38]. Activated SGK1 interacts with the WW domain of Nedd4-2 and phosphorylates it (Ser448 and 338), recruits and binds 14–3-3 protein, prevents the endocytosis and degradation of ENaC, and increases the activity of ENaC [39]. Our experiments demonstrated that the expression levels of the SGK1 signaling pathway-related proteins p-Nedd4-2, 14–3-3, and ENaC-a were significantly decreased in the decidua of URSA mice compared with controls.

Relevant studies have shown that the transcriptional level and activity of SGK1 in the mid-secretory endometrium of patients with RSA is reduced, which reduces the transcription and activity of ENaC, leading to a decrease in the absorption of intrauterine fluid and affecting the normal implantation of embryos [40]. Decreased levels of ENaC expression in placental tissue from pregnant women with preeclampsia (PE) may affect trophoblast migration and invasion, interfering with normal placenta formation [35]. Decreased SGK1 reduces the expression and activation of ENaC at the maternal–fetal interface, which mediates Na + currents involved in cell migration and proliferation, thereby affecting trophoblast invasion and proliferation, leading to miscarriage [40]. In vitro experiments confirmed that knockout of SGK1 or administration of SGK1-interfering agents reduces the viability of decidual stromal cells and decreases the expression of the decidualization marker gene PRL, resulting in abnormal decidua and placenta formation [41]. Likewise, the present study found low expression levels of PRLP and IGFBP-1 in the URSA group, suggesting impaired decidualization. In addition, the amount of serum PRL in the URSA group was significantly decreased compared with the controls. Therefore, based on the data in this paper combined with previous studies, we speculate that the SGK1/ENaC signaling pathway in decidual tissue is inhibited in individuals with URSA, resulting in insufficient decidualization and reduced expression of decidualization marker molecules, which in turn leads to the occurrence of URSA.

In recent years, SGK1 has been widely used as a therapeutic target for disease [42]. SGK1 is a key regulator in acute lung injury, and studies have shown that 17β-estradiol increases ENaC expression and inhibits inflammation through the PI3K/AKT/SGK1 signaling pathway, thereby relieving pulmonary edema to a certain extent. This suggests that SGK1 is expected to be an indirect target for improving ENaC function [43]. Furthermore, a lower copy number of the SGK1 gene is associated with poorer survival in glioblastoma multiforme, and an increase in SGK1 favors the survival of patients with glioblastoma multiforme [44]. Our study found that SGK1 and its signaling pathway-related proteins (p-Nedd4-2, 14–3-3 protein, ENaC-α), estrogen and progesterone and their receptors (E_2_, P, ERβ, PR), and decidualization markers (PRL, PRLR, IGFBP-1) were expressed at lower levels in unexplained recurrent miscarriage tissue. In addition, studies have shown that dydrogesterone is a commonly used drug for assisted reproductive technology, especially in the treatment of recurrent miscarriages [45]. This suggests that SGK1 may be a therapeutic target of dydrogesterone. In our study, mice treated with dydrogesterone had a reduced rate of embryo loss, indicating the therapeutic effect of dydrogesterone on URSA. The mouse experiments further confirmed that the expression level of SGK1 in the decidual tissue of the dydrogesterone-treated group was upregulated. In addition, the expression of signaling pathway-related proteins p-Nedd4-2, 14–3-3 protein, ENaC-α, and serum E_2_ and P, and ERβ and PR in the URSA + DQYT group were higher than those in the URSA group. This study suggests that SGK1 may serve as a therapeutic target of dydrogesterone, with dydrogesterone treatment increasing the levels of SGK1 and its pathway-related proteins. Additionally, it showed that the levels of decidualization-related molecules were upregulated in the dydrogesterone intervention group compared with the URSA group. Therefore, based on previous studies, we believe that estrogen and progesterone may mediate the SGK1/ENaC signaling pathway, inducing an impaired decidualization response and leading to the development of URSA, and this decidualization response can be mediated by the activator of SGK1, dydrogesterone intervention.

## Conclusions

**Fig. 6:**
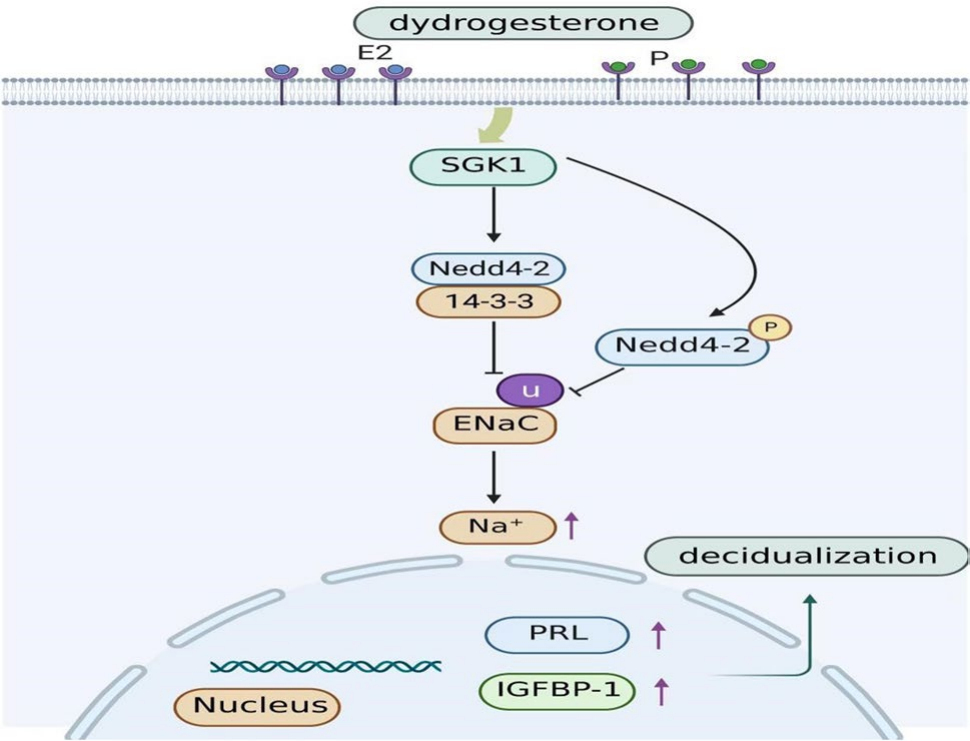
The summary of this study. Dydrogesterone promotes decidualization by increasing estrogen and progesterone levels and activating SGK1/ENaC signaling pathway

Taken together, these results suggest that in individuals with URSA, SGK1 is underexpressed and its signaling pathway is inhibited, which then leads to impaired decidualization and ultimately to the development of URSA (Fig. 6). In addition, dydrogesterone has a therapeutic effect on unexplained recurrent miscarriage. This is a preliminary exploration of the role of SGK1 as a target of dydrogesterone and provides a theoretical basis for the clinical treatment of URSA patients.

## Data Availability

The datasets used and/or analyzed during the current study are available from the corresponding author upon reason-able request.
